# Surgery

**Published:** 1893-12

**Authors:** 


### SURGERY.

EDITED BY DR. F. W. McRAE.

### TREATMENT OF SYPHILIS.

I have surely not the intention, in this correspondence, to speak to you of the last work of Professor Fournier upon the treatment of syphilis. It is a book to have, and one which one can scarcely analyze. Its appearance has freshened an old quarrel. It is well known that Professor Fournier believes that we must treat methodically and preventively every primary and secondary syphilis. Usually he prescribes to a patient who comes to see him at the beginning of his syphilis, a course of proto-iodide of mercury, in daily doses of six centigrams, to be taken for at least two months. Then this treatment is suspended for a month to six weeks, after which, no matter what has happened, whether the patient has or has not shown new developments, he has him take up the treatment by the proto-iodide again for about six weeks. Then there is a new period of repose for two or three months, renewed administration of the proto-iodide, new repose, and thus on until the third year of the disease. At this time he gives iodide of potassium in daily mean dose of three grammes, during periods of from one to six weeks, separated by intervals of rest, growing gradually longer as the time from the commencement of the disease increases. His treatment is thus a systematic one, very prolonged and intermittent. On the other hand, there are syphilographers in France, and Dr. Diday, of Lyons, is their venerated chief and dean, who maintain that we must never treat a syphilitic excepting when visible manifestations are present; that we must cease the impregnation with mercury as soon as there are no longer manifestations of the disease, for, according to their view mercury has no anti-syphilitic action in a preventive sense. This is the method called *opportuniste.*

You can understand how this debate is vigorously contested, and how important a thing it is that the question should once and for all be definitely settled. The appearance of Professor Fournier's book immediately called out the publication of an answer, in which Dr. Diday refutes the arguments of his adversary, and in which he shows, in the most spiritual manner, how, with a little dexterity, one can take the opposite side of any question and make statistics serve the purpose of proving any opinion. After reading most of the documents which have appeared on this point, one arrives at the conclusion, truly deplorable, that one cannot form a firm conviction, and that the two opinions are defensible, although, according to our views, that professed by Fournier seems to repose upon a more scientific base. However, in reasoning from another point of view, it seems to us that, when in doubt, one should not abstain from treatment, since the doses of medicine that are given at the present day are, in the immense majority of cases, in no wise injurious, and they should be continued with; for, if they are omitted, severe accidents may occur, and there will be no excuse.—*L. Brocq, in Jour. Cutaneous Dis.*

---

### TWO DEATHS BY CHLOROFORM—INTERESTING STATISTICS REGARDING ANESTHESIA.

Muschenkow, of Russia, writes in the *Chirurgische Annalen* of 1893: He operated upon a boy eight years old, suffering from tuberculous osteomyelitis. Patient otherwise in normal condition. The entire operation, including scraping, thermocautery and bandaging, lasted ten minutes. The operator was washing his hands, when patient becoming deathly pale, pupils dilated, death ensued. Four grammes of chloroform were used; the same kind was administered previously without ill-effects.

Methods of resuscitation tried: artificial respiration, ether, friction, electricity.

A. Waganow also reports a case in the *Chirurgische Annalen* of 1893. A ten-year-old boy was operated upon for "stone in the bladder," in the clinic of Professor Szintzin. Heart found to be normal before the operation.

Fifteen minutes after the beginning of the administration of chloroform, four grammes having been used, patient's pulse goodsuddenly cessation of heart's action and respiration occurred. With the syncope, evacuations of bowels and bladder and priapism was noticed.

The following methods tried: Sylvester's artificial respiration, subcutaneous injections of 300 grammes of salt, electricity to the phrenic nerve, tincture valerian, ether and hypodermatic injections of caffeine natro salicyl, heat, mustard, etc. Time, one and a half hours.

Egbert Braatz, a German, remarks with reference to these cases: Chloroform is used exclusively in Russia, and to a very large extent in Germany. It may safely be predicted, that ether, which is by far the less dangerous, will be used everywhere and by all physicians having the safety of their patients at heart.

Very interesting statistics regarding the different anesthetics were placed before the last (twenty-second) Congress of the German Surgical Association.

These statistics comprise 157,815 cases of anesthesia, with fifty-three deaths.

The ratio of deaths for the different anesthetics is as follows:

*Forty-six deaths* in 130,609 cases by *Chloroform*, or 1 :2839.
*No death* in 14,506 cases by *Ether*.
*One death* in 4,118 cases by combinations of *Chloroform and Ether*.
*No death* in 3,450 cases by *Chloroform, Ether and Alcohol*.
*One death* in 4,538 cases by *Bromide of Ethyl*.
*Three deaths* in 597 cases by *Pental*, or 1 : 199.

—*Translated by Dr. L. Amster, Atlanta.*

---

### ARSENIC IN EPITHELIOMA.

Lassar, *Berl. klin. Woch.*, *Br. Med. Jour.*, reports his success with arsenic administered internally in four cases of epithelioma affecting various parts of the face. Case 1 was a man, aged fifty, with three large swellings occupying one orbit, the nose and the chin respectively. Microscopic evidence showed epithelial cells, spindle cells and alveolar structure. Immediately after the administration of arsenic, the three growths gradually diminished by drying up, involution, and cicatrization, until the youngest growth had disappeared, and the second one cicatrized. The largest and oldest growth occupying nearly the whole of the orbit, showed little change, and owing to the suggested excision of the eyeball, the patient withdrew from treatment, and is believed to have died subsequently. In a second case, that of a woman of advanced age with a smaller growth on the nose, a great reduction in size took place, and the patient, being satisfied, also ceased to attend.

The author now resolved to adopt the same measure with recent growths instead of at once resorting to the knife. The first patient had on one cheek a growth equal to half a walnut, which had taken six or eight months in developing. Only a slight erosion of the surface was present. The diagnosis was confirmed microscopically, and arseniate of potash was administered three times daily for two months, when the growth had shrunk and cicatrized. The next patient was a man with a similar growth of three months' standing on the left ala nasi, the condition and proofs being the same. Fowler's solution was given internally, accompanied at first by subcutaneons injections. These being painful were discontinued, and in two months complete disappearance with cicatrization followed. The author admits the small number of cases experimented on, but lays stress on the striking and indisputable results. Illustrations of the patients at various times and of the microscopic sections are given.—*Canada Lancet.*

---

### RECURRENCE OF CARCINOMA OF THE BREAST.

Doubtless every practitioner who has had any considerable experience in the removal of carcinomatous tumors of the breast has had occasion to be discouraged with the results of the operative treatment, however thorough this may have been. Early and complete extirpation of the growth removes the local disease, but does not remove the susceptibility or predisposition to the disease; and surgeons always speculate as to the length of time a patient on whom they have operated for ablation of a cancerous tumor will enjoy immunity from the disease.

Dr. Frederic S. Dennis, of New York, who has made an exhaustive study of the subject, affirms that surgical interference can prevent recurrence in 90 per cent. of the cases, if, in addition to the complete removal of the breast, the axillary glands and fatty tissue are also removed. He says the liability to recurrence is reduced by an early diagnosis and operation, and that in a large majority of cases the diagnosis can be made before glandular infection has taken place; he adds that if within six months from its incipiency the tumor is removed, together with the axillary glands, fatty tissues, pectoral fascia, perimammary fat, and paramammary areola tissue, the chances of recurrence will be small. The operation should be so thorough as to leave out of consideration altogether the question of flaps to cover the wound.

Dennis reports a series of seventy-one cases in which he performed the complete operation, with no mortality. The radical operation removes cancerous cells that form the foci for recurrences. These cancer cells may be found outside the limits of the breast gland, lodged in the adjacent mammary region, whither they have been carried by the lymphatic current. Cancerous masses have been found upon and in and under the pectoral fascia, as well as in the neighboring muscles. Here removal of the breast is inadequate to remove the entire disease.

The recurrence of carcinoma of the breast is influenced by the histological character of the carcinoma itself. According to Dennis, the more typical the structure, the better the prognosis; the more atypical the structure, the more unfavorable the prognosis. The appearance simultaneously of carcinoma in both breasts—a condition which exists in five per cent. of all cases—also affects the chances of permanent recovery. There seems further, as Dennis shows, to be less malignancy in carcinoma affecting the early stages of obsolescence of the gland than when the gland has fully completed its degenerative changes. In other words, the prognosis is more unfavorable for old people than for persons in the full vigor of life. "The nearer the gland is to a healthy functional activity, the less likely is it to assume malignancy."—*Med. Age.*

